# Chlorogenic Acid Attenuates Doxorubicin-Induced Oxidative Stress and Markers of Apoptosis in Cardiomyocytes via Nrf2/HO-1 and Dityrosine Signaling

**DOI:** 10.3390/jpm13040649

**Published:** 2023-04-10

**Authors:** Betul Cicek, Ahmet Hacimuftuoglu, Yesim Yeni, Betul Danisman, Mustafa Ozkaraca, Behzad Mokhtare, Mecit Kantarci, Marios Spanakis, Dragana Nikitovic, Georgios Lazopoulos, Konstantinos Tsarouhas, Aristidis Tsatsakis, Ali Taghizadehghalehjoughi

**Affiliations:** 1 Department of Physiology, Faculty of Medicine, Erzincan Binali Yildirim University, 24100 Erzincan, Turkey; 2 Department of Medical Pharmacology, Faculty of Medicine, Ataturk University, 25240 Erzurum, Turkey; 3 Department of Medical Pharmacology, Faculty of Medicine, Malatya Turgut Ozal University, 44210 Malatya, Turkey; 4 Department of Biophysics, Faculty of Medicine, Ataturk University, 25240 Erzurum, Turkey; 5 Department of Pathology, Faculty of Veterinary, Sivas Cumhuriyet University, 58140 Sivas, Turkey; 6 Department of Radiology, Faculty of Medicine, Ataturk University, 25240 Erzurum, Turkey; 7 Department of Forensic Sciences and Toxicology, Faculty of Medicine, University of Crete, 71003 Heraklion, Greece; 8 Dragana Nikitovic, Laboratory of Histology-Embryology, School of Medicine, University of Crete, 71003 Heraklion, Greece; 9 Department of Cardiac Surgery, University General Hospital of Heraklion, Medical School, University of Crete, 71003 Heraklion, Greece; 10 Department of Cardiology, University General Hospital of Larissa, 41110 Larissa, Greece; 11 Department of Medical Pharmacology, Faculty of Medicine, Bilecik Seyh Edebali University, 11230 Bilecik, Turkey

**Keywords:** Nrf2/HO-1, cardiotoxicity, doxorubicin, chlorogenic acid, oxidative stress

## Abstract

(1) Background: Doxorubicin (DOX) is extensively used for cancer treatments; however, its clinical application is limited because of its cardiotoxic adverse effects. A combination of DOX and agents with cardioprotective properties is an effective strategy to ameliorate DOX-related cardiotoxicity. Polyphenolic compounds are ideal for the investigation of novel cardioprotective agents. Chlorogenic acid (CGA), an essential dietary polyphenol found in plants, has been previously reported to exert antioxidant, cardioprotective, and antiapoptotic properties. The current research evaluated CGA’s in vivo cardioprotective properties in DOX-induced cardiotoxicity and the probable mechanisms underlying this protection. (2) Methods: CGA’s cardioprotective properties were investigated in rats that were treated with CGA (100 mg/kg, p.o.) for fourteen days. The experimental model of cardiotoxicity was induced with a single intraperitoneal (15 mg/kg i.p.) injection of DOX on the 10th day. (3) Results: Treatment with CGA significantly improved the DOX-caused altered cardiac damage markers (LDH, CK-MB, and cTn-T), and a marked improvement in cardiac histopathological features accompanied this. DOX downregulated the expression of Nrf2/HO-1 signaling pathways, and the CGA reversed this effect. Consistently, caspase-3, an apoptotic-related marker, and dityrosine expression were suppressed, while Nrf2 and HO-1 expressions were elevated in the cardiac tissues of DOX-treated rats after treatment with the CGA. Furthermore, the recovery was confirmed by the downregulation of 8-OHdG and dityrosine (DT) expressions in immunohistochemical findings. (4) Conclusions: CGA demonstrated a considerable cardioprotective effect against DOX-induced cardiotoxicity. One of the possible mechanisms for these protective properties was the upregulation of the Nrf2/HO-1-dependent pathway and the downregulation of DT, which may ameliorate oxidative stress and cardiomyocyte apoptosis. These findings suggest that CGA may be cardioprotective, particularly in patients receiving DOX-based chemotherapy.

## 1. Introduction

Doxorubicin (DOX) is a cytotoxic anthracycline antibiotic derived from Streptomyces that is administered for the therapy of many types of malignancies, such as solid tumors, lymphomas, leukemia, and lung and breast cancer [1,2]. Despite its effectiveness, its clinical application shows many adverse effects, particularly dose-dependent and cumulative cardiotoxicity resulting in cardiac dysfunction, heart failure, and eventually death [3,4,5,6]. The underlying mechanisms responsible for DOX-induced cardiotoxicity are complex and multifactorial, related to the disturbance of intracellular calcium homeostasis, DNA/RNA/protein synthesis inhibition, reactive oxygen species (ROS) accumulation, and necroptosis induction [7,8,9,10]. Previous studies have shown that excessive ROS production leads to oxidative damage to biological macromolecules, including proteins, DNA, and lipids, and depletes antioxidant enzymes [10,11,12,13]. Moreover, DOX-evoked oxidative damage directly triggers the intrinsic mitochondria-dependent apoptotic pathway in cardiomyocytes and, in turn, leads to the death of myocytes [14]. Furthermore, similar mechanisms have been identified in cardiotoxicity induced by other xenobiotics, including pesticides and nanoparticles [15,16,17,18].

Accumulating evidence suggests that the down-regulation of the nuclear factor erythroid-derived 2-like 2 (Nrf2)/heme oxygenase-1 (HO-1) signaling pathway is responsible for DOX-evoked myocardial damage [19]. In cases of augmented ROS production, Nrf2 travels to the nucleus, binds to the antioxidant response element (ARE) to form a complex of Nrf2-ARE, and then initiates the process of transcription of antioxidative genes and their proteins such as hemeoxygenase-1 (HO-1), superoxide dismutase (SOD), catalase (CAT), and glutathione peroxidase (GSH-Px) in order to detoxify the accumulated free radicals [20,21]. Hence, disruption of Nrf2/HO-1 signaling is thought to be a principal reason for DOX-related cardiotoxicity. For instance, Nordgren et al. reported that Nrf2 signaling and Nrf2-related antioxidant responses were strongly abrogated in cardiac tissue in DOX-treated rats [22]. Moreover, other reports confirm that DOX significantly reduces the expression of Nrf2, resulting in oxidative damage, cardiomyocyte apoptosis, and cardiac dysfunction in rats [23]. Therefore, activation of Nrf2/HO-1 signaling is regarded as a probable and effective strategy to overcome DOX-evoked cardiotoxicity.

On the other hand, 3,3′-dityrosine, also known as dityrosine (DT), plays the essential role of oxidative perturbation, which has a principal function in the progression of cardiac dysfunction [24]. DT leads to oxidative modifications in proteins, and hydroxyl radicals and lipid hydroperoxides promote DT action [24,25]. Although the mechanisms mediated by DT in DOX-related cardiotoxicity remain largely unknown, Benzer et al. demonstrated that DOX-related cardiac damage is associated with a marked rise in DT expression concomitant with an elevation of oxidative damage [26]. Concerning the essential roles of oxidative perturbation and apoptosis in DOX-related cardiotoxicity, as mentioned above, we speculated that DT might be involved in regulating Nrf2/HO-1 signaling associated with DOX-evoked cardiac damage. 

Numerous studies have proposed that the bioactive compounds found in natural products can alleviate DOX-related cardiotoxicity [27,28,29]. Thus, it is logical to investigate the plant-derived natural compounds in order to reduce the DOX-associated cardiotoxic adverse effects and elevate the DOX chemotherapeutic impact. Among these bioactive compounds, dietary polyphenols, especially polyphenolic acids, attract scientific attention regarding their biological effects and potential pharmacological utilization [12,30]. Chlorogenic acid (3,4-dihydroxycinnamate, CGA) is the major polyphenolic isomer among caffeoylquinic acid isomers found in many plants, including *Crataegus monogyna*, Vaccinium angustifolium, and Eupatorium perfoliatum, and is particularly abundant in coffee [27]. CGA has many essential and therapeutic functions such as antioxidant, hepatoprotective, cardioprotective, anti-inflammatory, anti-apoptotic, and free radical scavenging, while it improves immune regulation [30]. Interestingly, despite the growing number of findings supporting the protective role of CGA in preclinical cardiac disease models, no study on the effect of CGA on DOX-induced cardiotoxicity in rats has been carried out. Therefore, the present study aimed to investigate the cardioprotective effects of CGA against DOX-induced myocardial toxicity and to elucidate whether this action was associated with the regulation of Nrf2/HO-1 and DT signaling and, consequently, with myocardial oxidative stress suppression.

## 2. Materials and Methods

### 2.1. Chemicals and Reagents

DOX was bought as Adrimisin^®^ (50 mg/25 mL injectable solution) from Saba Pharmaceuticals (Istanbul, Turkey). CGA (Cas Number #327-97-9) and Tris hydrochloride (Tris–HCl; Cas Number #1185-53-1) were obtained from Merck (Darmstadt, Germany). Sevoflurane anesthesia (Sevorane liquid, 100%) was obtained from Abbott Laboratory (Istanbul, Turkey). CK-MB (Cat.No.:E-EL-R1327), LDH (Cat.No.:E-BC-K046-M), cTn-T (Cat.No.: E-EL-R0151), malondialdehyde (MDA; Cat.No.:E-EL-0060), GSH(Glutathione; Cat.No.:E-EL-0026), GSH-PX (Cat.No.:E-BC-K096-S), SOD (Cat.No.:E-BC-K019-S), and CAT (Cat.No.:E-BC-K031-S) were bought from Elabscience (Houston, TX, USA). Hematoxylin and eosin (H&E; Cat.No.:1051750500) was obtained from Merck (Darmstadt, Germany). 8-hydroxy-2′-deoxyguanosine 8-OhDG (Cat.No.:ab48508), 4-hydroxynonenal (4-HNE; Cat.No.: ab46545), and dityrosine (DT; Cat.No.: ab243067) primary antibodies were obtained from Abcam (Boston, MA, USA). Horseradish peroxidase (HRP) (Cat.No.: TP-125-HL) was bought from Thermo Fisher (Istanbul, Turkey).

### 2.2. Animals

Male Wistar Albino rats (230–250 g) were procured from the Ataturk University Experimental Research and Application Center (Erzurum, Turkey). The animals were housed under standard conditions (24 ± 1 °C and a 12:12 h dark/light cycle) with free access to food and water. 

### 2.3. Experimental Design

**Figure 1:**
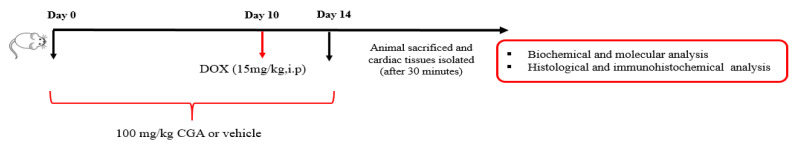
The flowchart of the experiment design.

Adult male Wistar Albino rats were randomly divided into four groups (n = 7) as follows: (1) control group: rats received vehicle (PBS daily for 14 days); (2) CGA group: rats received oral CGA at 100 mg/kg/day for 14 days; (3) DOX group: rats received a single dose of DOX at 15 mg/kg on the 10th day via intraperitoneal (i.p.); and (4) DOX  +  CGA group: rats were pretreated with oral CGA at 100 mg/kg/day for 14 days and received a single dose of DOX at 15 mg/kg on the 10th day via i.p. injection. The flowchart of the experiment is presented in Figure 1.

It is well established that acute DOX-induced cardiotoxicity is common in clinical practice, as reported in various studies [31,32], and predicts poor clinical outcomes [33]. A single dose of DOX (15 mg/kg, i.p.) was selected based on previously reported studies for its potential to establish acute cardiotoxicity in mice as well as rats [34,35]. The dose of CGA was selected on the basis of a previously reported study [36]. Meanwhile, the in vivo optimum dose of CGA was supported by a preliminary assay study with a limited number of animals (three animals/group). 

### 2.4. Serum Collection and Tissue Preparation

At the end of the study, animals went under anesthesia and blood samples were collected from the heart. The samples were centrifuged at 1500× *g* for 15 min at 4 °C; the serum was separated and stored at −20 °C until biochemical analysis. The animals were sacrificed under deep anesthesia after blood sampling. The chest was opened, and the cardiac tissues were promptly excised and washed in normal saline. Cardiac tissues were cut into two sections, homogenized with 0.1 M Tris–HCl buffer (pH 7.4), centrifuged at 10,000× *g* for 25 min at 4 °C, and the resulting supernatants were stored at −20 °C until the measurement of the cardiac levels of oxidative and inflammatory markers. The remaining cardiac samples were fixed in 10% formalin in phosphate buffer, processed through histological and histopathological methods, and then embedded in paraffin [37].

### 2.5. Estimation of Serum Biochemical Parameters

Serum creatine-kinase myocardial band (CK-MB) and lactate dehydrogenase (LDH) markers of myocyte necrosis were determined using commercial CK-MB and LDH kits by the auto-analyzer (Thermo Scientific, Waltham, MA, USA) according to the manufacturer’s instructions. A rat ELISA kit measured serum levels of cTn-T as a specific biomarker of cardiac injury, following the manufacturer’s guidelines [38].

### 2.6. Histopathological Examinations

The cardiac tissues were fixed overnight in 10% formalin and embedded in paraffin after routine alcohol-xylol follow-up procedures. In addition, 5μm sections were stained with H&E and evaluated semi-quantitatively randomly in 6 different areas in terms of mononuclear (Mn) cell infiltrations and hemorrhage under light microscopy as absent (-), mild (+), moderate (++), and severe (+++) [10,11]. Histopathological damage scores are presented in Table 1 [39]. The Kruskal-Wallis and Mann-Whitney U tests, non-parametric statistical tests, were used for the evaluation of the non-parametric data (absent, mild, moderate, and severe). The absent (-) value was presented as 0 in Table 1, the mild (+) value as 1, the moderate (++) value as 2, and the severe (+++) value as 3.

### 2.7. Lipid Peroxidation (LPO) Assay

The malondialdehyde (MDA) content was estimated to evaluate the peroxidation of lipids. Levels of MDA in heart tissue lysates were measured using a rat ELISA assay kit according to the manufacturer’s directions. Briefly, the samples and Biotinylated Detection Ab working solution were added to the well and incubated for 45 min at 37 °C. The well was then filled with HRP conjugate and incubated for 30 min. Finally, substrate reagent and stop solution were added, respectively, and the plate was read at 450 nm using the Multiskan™ GO Microplate Spectrophotometer reader (Thermo Scientific, Waltham, MA, USA). 

### 2.8. Estimation of Endogenous Antioxidants

The intracellular antioxidants in cardiac tissue, such as glutathione (GSH), glutathione peroxidase (GSH-PX), superoxide dismutase (SOD), and catalase (CAT), were quantified using commercial kits. GSH activity was measured according to the consumption of dithionitrobenzoic acid at 420 nm. GSH-PX activity was determined according to the consumption of nicotinamide dinucleotide phosphate acid at 412 nm. A SOD activity assay was carried out based on the inhibition of a superoxide radical reaction at 550 nm. Finally, CAT activity was estimated based on the consumption of hydrogen peroxide at 405 nm. 

### 2.9. Molecular Analysis

Total RNA was isolated from the cardiac tissue of rats with a TRIzol^®^ reagent by following the manufacturer’s instructions. RNA purity and concentrations were determined by measuring the 260/280-nm ratio. Total RNA was used for synthesizing complementary DNA (cDNA) using a high-capacity cDNA Reverse Transcription Kit. The Nrf2, HO-1, and caspase-3 gene expression levels (mRNA) were determined with Rotor-Gene Q (QIAGEN). GAPDH was used as the standard control protein in each sample. Primer sequences are shown in Table 2. The results obtained from our studies were expressed as a fold change in expression compared with expression in the control group, and CT values were automatically converted into 2^−ΔΔCt^ in the device. 

### 2.10. Immunofluorescent Staining

The 5 μm cross-sections were taken onto poly-L-lysine coated slides and then passed through the xylol and alcohol series. Subsequently, the cross-sections were washed with PBS and kept with 3% H_2_O_2_ for 10 min for endogenous peroxidase inactivation. The samples were incubated at 500 W in a microwave oven for 2 × 5 min with an antigen retrieval solution to retrieve the antigen from the cardiac tissues. After washing with PBS, the sections were incubated with 8-OhDG, 4-HNE, and DT primary antibodies overnight at 4 °C. Then, HRP was applied according to the manufacturer’s directions. Next, after counterstaining with Mayer’s hematoxylin, it was covered with entellan and examined under a light microscope. Immunopositivity in heart tissues was evaluated as none (0), mild (1), moderate (2), and severe (3).

### 2.11. Statistical Analyses

Data were analyzed with the Statistical Package for the Social Sciences (SPSS v.20, SPSS Inc., Chicago, IL, USA) and presented as mean ± SD. To determine normality and homogeneity, the Shapiro-Wilk and Levene tests were used. ELISA and RT-PCR results were analyzed by a one-way analysis of variance (ANOVA) followed by Tukey’s post hoc test (*p* < 0.05). Immunohistochemical findings were analyzed by Kruskal-Wallis with the Mann-Whitney U post hoc test (*p* < 0.05).

## 3. Results

### 3.1. Biochemical Myocardial Injury Markers

**Figure 2:**
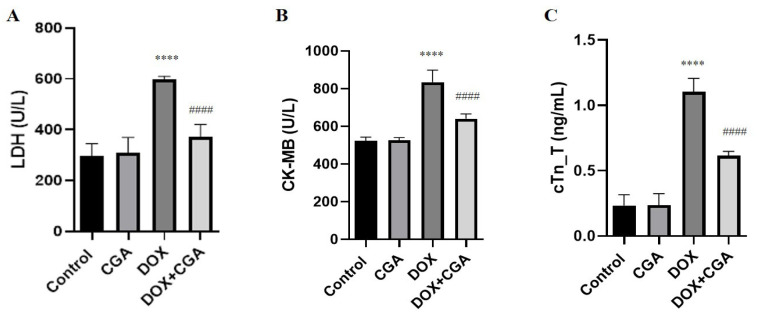
Serum cardiac damage marker levels. The serum levels of (**A**) LDH, (**B**) CK-MB, and (**C**) cTn-T were markedly decreased with CGA treatment. Data are presented as the means ± SD. **** *p* < 0.0001 vs. the control group, #### *p* < 0.0001 vs. the DOX group (n = 7). CGA: Chlorogenic acid (100 mg/kg); DOX: Doxorubicin (15 mg/kg).

The serum markers of myocardial damage (LDH, CK-MB, and cTn-T) are presented in Figure 2. DOX-induced cardiotoxicity was demonstrated via a significant elevation in serum LDH, CK-MB, and cTn-T concentrations in the DOX-treated group (596.9 ± 13.22 U/L; 834.9 ± 63.59 U/L; 1.10 ± 0.10 ng/mL, respectively) compared to control (295.2 ± 50.4 U/L; 522.5 ± 50.15 U/L; 0.23 ± 0.08 ng/mL, respectively) (*p* < 0.0001) and CGA-only treated groups (308.1± 61.66 U/L; 525.2 ± 36.85; 0.23 ± 0.08 ng/mL, respectively) (*p* < 0.0001). On the other hand, the serum levels of LDH (372 ± 48.92 U/L), CK-MB (640.7 ± 62.47 U/L), and cTn-T (0.61 ± 0.03 ng/mL) were significantly attenuated in DOX-exposed animals when the animals were pretreated with CGA compared to the DOX group (*p* < 0.0001). Furthermore, compared to the control group, CGA-only treated rats did not show any alterations in serum levels of LDH, CK-MB, and cTn-T levels (*p* > 0.05). 

### 3.2. CGA Prevents DOX-Induced Cardiac Injury

**Figure 3:**
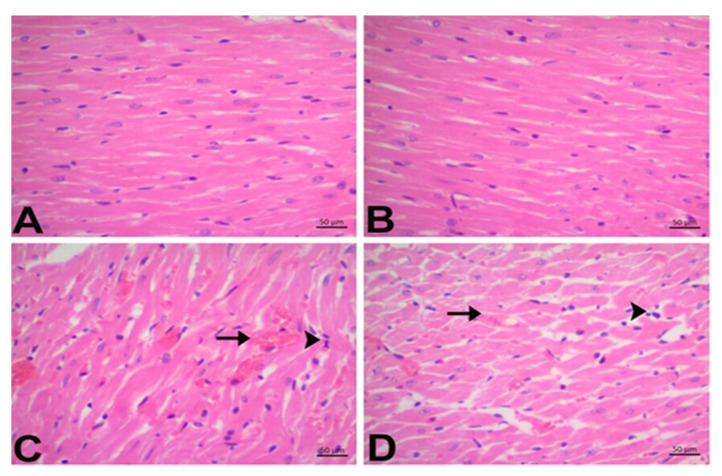
Effects of CGA on histopathological changes in DOX-treated cardiac tissue by H&E staining (magnification, ×400). (**A**) Control, (**B**) CGA, (**C**) DOX, and (**D**) DOX + CGA. Arrowhead: Mn cell infiltration score, Arrow: Hemorrhage. Data are expressed as the means ± SD. CGA: Chlorogenic acid (100 mg/kg); DOX: Doxorubicin (15 mg/kg). Arrowhead: Mn cell infiltration score, Arrow: Hemorrhage.

Histologically, the control group’s cardiac sections showed a normal histological structure. However, mononuclear (Mn) cell infiltration and hemorrhage were evident in DOX animals (*p* < 0.05). As shown in Figure 3D, oral pretreatment with CGA markedly alleviated Mn cell infiltration and hemorrhage compared to the DOX group (*p* < 0.05). Our findings also suggest that mononuclear (Mn) cell infiltration in the only CGA-treated group was higher than that in the control group; however, this was not statistically significant. Moreover, there was no hemorrhage in the CGA-only group (*p* > 0.05) (Figure 3, Table 3).

### 3.3. CGA Attenuates DOX-Induced LPO

**Figure 4:**
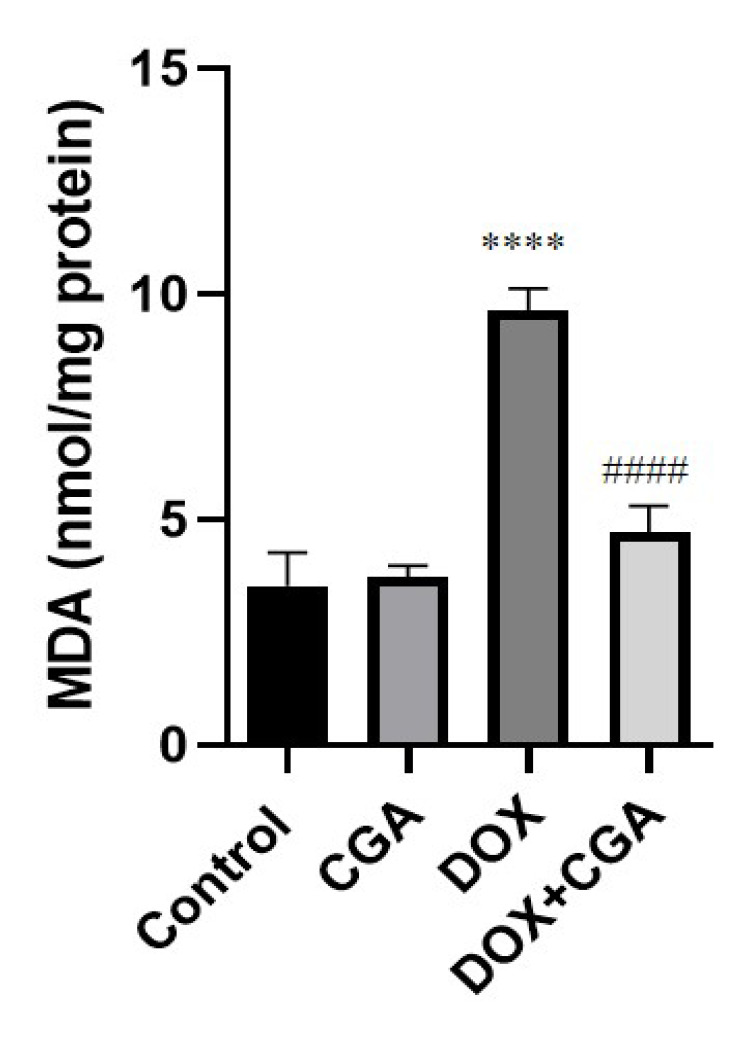
LPO marker (MDA) in cardiac tissue. CGA significantly reversed the DOX-related LPO via the reduction of MDA levels. Data are presented as the means ± SD. **** *p* < 0.0001 vs. the control group, #### *p* < 0.0001 vs. the DOX group (n = 7). CGA: Chlorogenic acid (100 mg/kg); DOX: Doxorubicin (15 mg/kg); MDA: Malondialdehyde.

As shown in Figure 4, induction of cardiotoxicity with DOX caused significantly increased MDA protein levels (9.64 ± 0.48 nmol/mg) in comparison with the control group (3.52 ± 0.73 nmol/mg protein) (*p* < 0.0001). CGA treatment in DOX-intoxicated rats significantly reduced MDA levels (4.71 ± 0.59 nmol/mg protein) in comparison with the DOX group (*p* < 0.0001), suggesting the possible antioxidant role of CGA against myocardial lipid peroxidation (LPO) induced by DOX. In Figure 4, no significant differences were evident between CGA-only treated and control rats (*p* > 0.05). 

### 3.4. CGA Alleviates DOX-Induced Oxidative Stress

**Figure 5:**
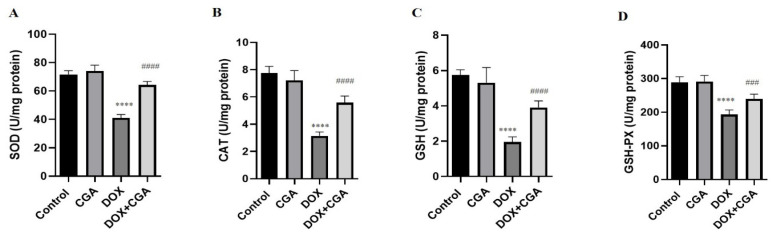
Oxidative stress markers in cardiac tissue. CGA significantly reversed the DOX-related oxidative stress via the elevation of (**A**) SOD, (**B**) CAT, (**C**) GSH-PX, and (**D**) GSH levels. Data are presented as the means ± SD. **** *p* < 0.0001 vs. the control group, ### *p* < 0.001, #### *p* < 0.0001 vs. the DOX group (n = 7). CGA: Chlorogenic acid (100 mg/kg); DOX: Doxorubicin (15 mg/kg); SOD: Superoxide dismutase; CAT: Catalase; GSH: Glutathione; GSH-PX: Glutathione peroxidase.

As shown in Figure 4, induction of cardiotoxicity with DOX significantly decreased SOD (41.12 ± 2.32 U/mg protein), CAT (3.14 ± 0.28 U/mg protein), GSH (1.95 ± 0.29 U/mg protein), and GSH-PX (193.2 ± 13.63 U/mg protein) levels in comparison with the control group (71.67 ± 2.59 U/mg protein; 7.76 ± 0.48 U/mg protein; 5.74 ± 0.30 U/mg protein; 289.2 ± 16.44 U/mg protein) (*p* < 0.0001). Administration of CGA in DOX-intoxicated rats significantly elevated SOD (64,25 ± 2,46 U/mg protein, *p* < 0.0001), CAT (5.57 ± 0.49 U/mg protein, *p* < 0.0001), GSH (3.91 ± 0.37 U/mg protein, *p* < 0.0001), and GSH-PX (239.5 ± 14.22 U/mg protein, *p* < 0.001) levels in comparison with DOX-treated rats. Statistical analysis findings demonstrated no marked difference between CGA-only treated and control rats (*p* > 0.05) (Figure 5). 

### 3.5. CGA Activates the Nrf2/HO-1 Pathway in DOX-Treated Cardiac Tissue

**Figure 6:**
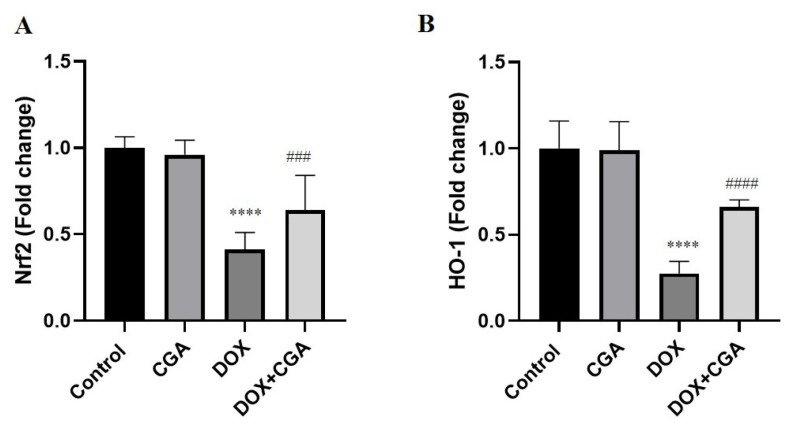
(**A**) Nrf2 and (**B**) HO-1 signaling pathway in cardiac tissue. The expression levels of Nrf2 and HO-1 were elevated with CGA treatment. Data are presented as the means ± SD. **** *p* < 0.0001 vs. the control group, ### *p* < 0.001, #### *p* < 0.0001 vs. the DOX group (n = 7). CGA: Chlorogenic acid (100 mg/kg); DOX: Doxorubicin (15 mg/kg); Nrf2: Nuclear factor erythroid 2–related factor 2; HO-1: Heme Oxygenase 1.

Cellular responses to oxidative damage are partially modulated by the redox-sensitive transcription factors Nrf2 and HO-1. As shown in Figure 6A,B, the expression levels of Nrf2 and HO-1 in the cardiac tissue of rats significantly decreased in the DOX group (0.41 ± 0.10-fold change; 0.28 ± 0.07-fold change, respectively) in comparison with the control group (1.00 ± 0.06-fold change; 1.00 ± 0.16 fold change, respectively) (*p* < 0.0001). Treatment with CGA in DOX-intoxicated rats markedly increased Nrf2 (0.64 ± 0.20-fold change; *p* < 0.001) and HO-1 (0.66 ± 0.04-fold change; *p* < 0.0001) expression levels in comparison with DOX-treated rats. Statistical analysis results also showed no significant difference in levels of Nrf2 and HO-1 expression among CGA-only treated and control rats (*p* > 0.05).

### 3.6. CGA Reduces DOX-Induced Caspase-3 Expression

**Figure 7:**
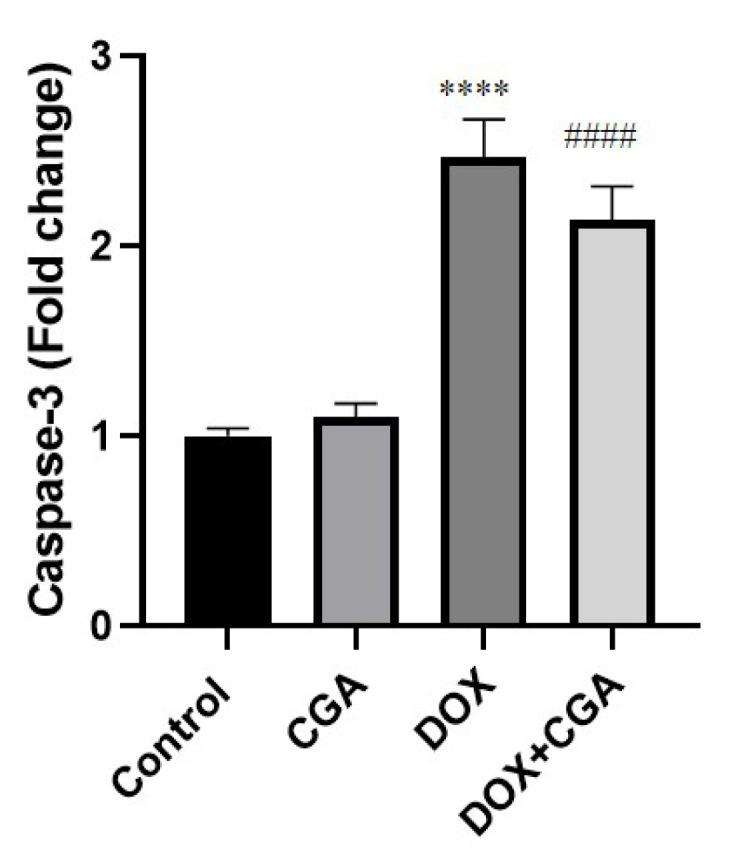
Apoptotic marker in cardiac tissue. The caspase-3 mRNA expression levels were significantly decreased with CGA treatment. Data are presented as the means ± SD. **** *p* < 0.0001 vs. the control group, #### *p* < 0.0001 vs. the DOX group (n = 7). CGA: Chlorogenic acid (100 mg/kg); DOX: Doxorubicin (15 mg/kg).

To evaluate the effect of CGA in a probable DOX-induced apoptotic pathway, we examined expression levels of caspase-3 in cardiac tissues. Our results showed that mRNA levels of caspase-3 in cardiac tissue were markedly elevated in DOX-intoxicated rats (2.46 ± 0.20-fold change) compared to the control group (1.00 ± 0.04-fold change) (*p* < 0.0001) (Figure 6). Following treatment with CGA in DOX-intoxicated rats, mRNA expression of caspase-3 (2.13 ± 0.18-fold change) declined in comparison to the DOX group (*p* < 0.0001). Figure 6 also indicated no significant differences in caspase-3 mRNA expression levels between CGA-only treated and control rats (*p* > 0.05) (Figure 7). 

### 3.7. Immunohistochemical Findings

**Figure 8:**
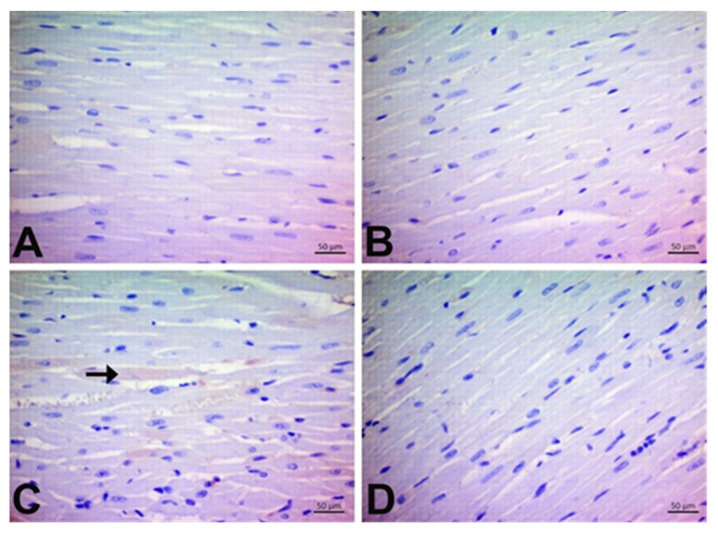
Immunohistochemical staining of 4-HNE in experimental groups in cardiac tissue (×400). 4-HNE expression levels were significantly elevated in DOX-treated rats, while CGA-treated rats demonstrated a significant decline in 4-HNE expression. Arrowhead: Intensity of immunoreactivity. (**A**) Control, (**B**) CGA, (**C**) DOX, and (**D**) DOX + CGA. Data are presented as the means ± SD. CGA: Chlorogenic acid (100 mg/kg); DOX: Doxorubicin (15 mg/kg); CGA: Chlorogenic acid (100 mg/kg); DOX: Doxorubicin (15 mg/kg); 4-HNE: 4-Hydroxynonenal.

**Figure 9:**
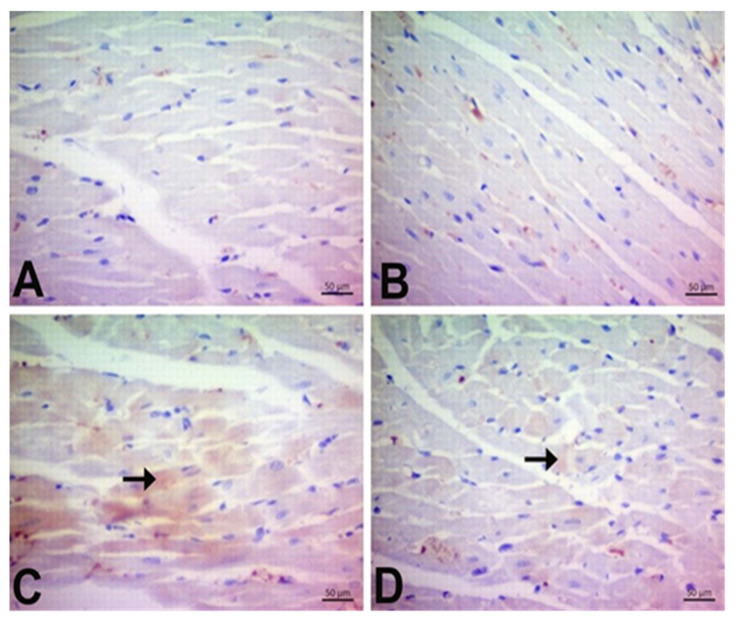
Immunohistochemical staining of 8-OHdG in experimental groups in cardiac tissue (×400). The 8-OHdG expression levels were significantly elevated in DOX-treated rats, while CGA-treated rats demonstrated a significant decline in 8-OHdG expression. Arrowhead: Intensity of immunoreactivity. (**A**) Control (**B**) CGA (**C**) DOX (**D**) DOX + CGA. Data are presented as the means ± SD. CGA: Chlorogenic acid (100 mg/kg); DOX: Doxorubicin (15 mg/kg).

**Figure 10:**
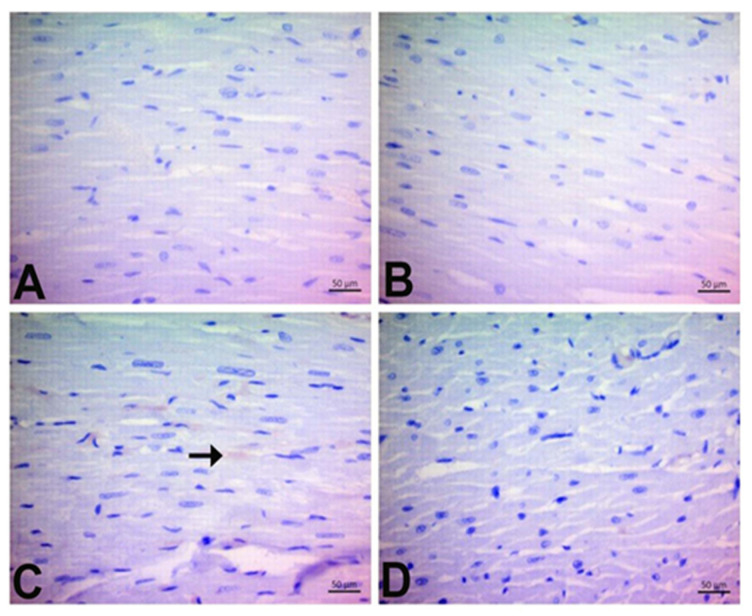
Immunohistochemical staining of DT in experimental groups in cardiac tissue (×400). DT expression levels were significantly elevated in DOX-treated rats while CGA-treated rats demonstrated a significant decline in DT expression. Arrowhead: Intensity of immunoreactivity. (**A**) Control, (**B**) CGA, (**C**) DOX, and (**D**) DOX + CGA. Data are presented as the means ± SD. CGA: Chlorogenic acid (100 mg/kg); DOX: Doxorubicin (15 mg/kg); DT: dityrosine.

In the cardiac sections, 4-HNE, 8-OHdG, and DT immunoreactivity were assessed (Figure 8, Figure 9 and Figure 10, respectively, and Table 4). No 4-HNE, 8-OHdG, or DT staining was observed in the control group. DOX treatment caused a pronounced elevation in the expression of 4-HNE, 8-OhDG, and DT in rat cardiac tissues (*p* < 0.05). Our results demonstrate that the number of 4-HNE, 8-OHdG, and DT-positive cells was reduced in DOX + CGA treated rats compared to the DOX group (*p* < 0.05). On the other hand, there are no significant differences in the expression levels of 4-HNE, 8-OHdG, and DT between CGA-only treated and control rats.

## 4. Discussion

In the present study, we found that pretreatment with CGA protected rats from DOX-induced cardiotoxicity, ameliorated myocardial tissue damage, inhibited the oxidative response, and alleviated myocardial apoptosis. Pretreatment, which was selected at the study planning stage, is a valid approach to the problem of chemotherapy-linked cardiotoxicity. In clinical practice, specific patient groups are known to be in greater danger of developing cardiotoxicity; for example, patients with hypertension, females, and patients planning to receive a high cumulative dose of cardiotoxic drugs such as DOX [40,41].

The DOX-induced elevation of serum levels of LDH, CK-MB, and cTn-T, which indicate the severity of cardiac damage, was significantly reversed by CGA. The protective effect of CGA may be associated with the activation of the Nrf2/HO-1 signaling pathway and amelioration of DT expression in DOX-treated rats. Previous studies showed that DOX administration leads to the accumulation of free radicals in cardiac tissue, damaging intracellular components and cell membranes [13]. Damage to the cardiac muscle cells’ membrane results in the release of LDH, CK-MB, and cTn-T in the blood circulation. LDH and CK-MB are ordinarily present in the cellular compartment, but during myocardial damage, they leak out into the circulation due to the disintegration of cardiomyocytes [42].

cTn-T is a specific marker of myocardial cell injury. This contractile protein is normally not present in the blood but only in cardiac tissue and is released during myocardial damage [43]. DOX-induced oxidative damage to the myocardium leads to cell membrane permeation or rupture, which causes leakage of cardiac enzymes into the circulation [19].

In the current study, elevated levels of LDH, CK-MB, and cTn-T detected in the serum of DOX-treated rats clearly indicate that DOX leads to cardiac damage. These cardiac enzymes’ increased activity agrees with previous reports [3,19]. However, treatment with CGA markedly reduced LDH, CK-MB, and cTn-T levels in the serum of DOX-treated rats, suggesting the protective effects of CGA in reversing DOX cardiac injury. The histopathological analysis demonstrating the Mn cell infiltration and hemorrhage in the cardiac tissue of DOX-treated rats is in accordance with previous reports [14,19]. CGA treatment ameliorated the deleterious DOX effects in the myocardium, as confirmed by cardiac tissue damage markers. The said migration of Mn cells towards the myocardial regions primarily affected by doxorucin treatment, along with the hemorrhages observed, are often responsible for a regional increase in connective tissue or can cause morphological changes in myocardial cells as a result of these processes. However, no remarkable increase in connective tissue was observed in all experimental rats during the study period.

Many studies have reported that disturbed redox balance associated with the production of excessive ROS is correlated with the mechanism of DOX-related cardiotoxicity [10,22,44]. Elevated levels of ROS impair both DNA and protein function and lead to lipid oxidation of cell membranes, leading to cardiac dysfunction and cell death [45]. The biomolecules affected earliest by oxidative stress are lipids. As a consequence of LPO, aldehydes, including MDA and 4-HNE, are formed [23]. Previous research reported that MDA and 4-HNE could directly weaken cardiac contractile function, showing the impact of oxidative stress and DOX-related cardiac damage on the myocardium [46]. In the current study, significant increases in levels of MDA and the expression of 4-HNE were found in the cardiac tissue of DOX-treated rats. The results are in accordance with previous reports indicating that elevated levels of lipid peroxides in DOX-treated rats result in ROS-induced oxidative cell damage [23,26,47]. Administration of CGA resulted in a marked reduction of MDA and 4-HNE in DOX-treated rats. Li et al. have shown that CGA might preclude the propagation of the LPO process by scavenging ROS, resulting in the control of oxidative injury and cardiac tissue protection in mice with myocardial infarction [48].

The elevated level of LPO in the cardiac tissue is mainly attributed to oxidative damage, which is related to the reduced levels of enzymatic and non-enzymatic antioxidants such as SOD, CAT, GSH-PX, and GSH [19]. In this study, DOX treatment weakened the innate antioxidant defense mechanism, as evident from the consumption of GSH and the reduced activities of SOD, CAT, and GSH-PX connected with elevated LPO and oxidative stress in the rats’ cardiac tissue. Myocardial oxidative damage induced by DOX via excessive generation of ROS has been previously demonstrated in many studies [8,11,35]. In this report, CGA prevented DOX-induced oxidative injury by increasing SOD, CAT, and GSH-PX activities and replenishing GSH content concomitant with the reduction of LPO in cardiac tissue. The current study is in accordance with the findings of Akila et al., who showed that CGA ameliorated oxidative stress in the experimental setting of isoproterenol-induced myocardial oxidative stress and damage in rats [49]. It is evident that most of the myocardium-salvaging therapies in the setting of cardiooncology focus on oxidative stress suppression, which is at the same time necessary for the primary function of chemotherapy, the killing of cancer cells, but achieving this specific balance, for the time being, represents a valid approach for counteracting cardiotoxicity [50].

Furthermore, oxygen radicals can also damage DNA. It is well known that one of the most important parameters for evaluating oxidative DNA damage in biological systems is 8-OHdG. A marked elevation was observed in the 8-OHdG expression levels in the cardiac tissue of DOX-treated rats [26]. In accordance with our results, it was previously reported that DOX evoked DNA damage with increased 8-OHdG expression [51]. Oxidative stress could be the first stressor and initial biochemical pathway implicated in the process of doxorubicin cardiotoxicity and the subsequent myocardial functional decline. In our study, the 8-OHdg (IHC staining) is used for the actual investigation and presentation of DNA fragmentation. As is known, DNA fragmentation is also seen in the last step of apoptosis. The CGA treatment downregulated the 8-OHdG levels. These findings demonstrated the protective effects of CGA on the antioxidant mechanism and displayed its role in scavenging free radicals. However, no studies have evaluated CGA effects on 8-OHdG in cardiovascular disease. On the other hand, Hada et al. reported that CGA suppressed the high expression level of 8-OHdG and prevented DNA destruction, thus protecting endothelial cells from senescence in an experimental in vitro model [52].

The Nrf2/HO-1 signaling pathway is critical in scavenging ROS and extenuating DOX-induced oxidative cardiac damage [19,44]. In previous reports, reduced expression levels of Nrf2/HO-1 signaling were closely linked to exacerbations of DOX-induced cardiotoxicity and cardiac dysfunction [53]. In the current study, we found that DOX downregulated Nrf-2 and its target gene expression, HO-1, in harmony with the decrease of antioxidant enzyme levels, including SOD, CAT, GSH-PX, and GSH. These results demonstrated that DOX treatment inhibits the activation of Nrf2/HO-1 in cardiac tissue, thus supporting the concept that pharmacological or phytochemical activation of Nrf2/HO-1 could mitigate DOX-related cardiac damage, including strengthening the endogenous antioxidative function [52]. Previous studies reporting the beneficial effects of CGA on oxidative damage inspired us to investigate CGA administration in DOX-related cardiotoxicity [30]. CGA treatment prevented DOX-induced myocardial damage by regulating Nrf2/HO-1 expression and myocardial antioxidant status. To our knowledge, the current study is the first to examine CGA’s role in Nfr2/HO-1 signaling in cardiovascular disease. Nevertheless, Zheng et al. had previously demonstrated the CGA protective effects against oxidative stress by activating the Nrf2/HO-1 axis in hypoxia-ischemia brain injury in neonatal rats [54].

Recently, an increasing number of studies have reported that programmed cell death, or cellular apoptosis, is necessary for regulating DOX-related cardiac damage [26]. At the same time, DOX-evoked oxidative damage results in the activation of an “intrinsic”, mitochondria-dependent apoptotic pathway that directly triggers, in excess, cardiac apoptosis [47]. Caspase-3 is the major executor of apoptosis in cardiac damage [3]. Caspase-3 is required for the characteristic features of apoptosis and is indispensable for apoptotic chromatin condensation and DNA fragmentation in all cell types examined. Thus, caspase-3 is essential for the initiation of the processes associated with the dismantling of the cell and the formation of apoptotic bodies. In agreement with previous reports, the mRNA levels of caspase-3 were elevated in the DOX-treated rats, indicating that oxidative stress may lead to cardiomyocyte apoptosis by activating the mitochondrial apoptosis pathway [13,34]. Furthermore, Al-Rasheed et al.’s [55] research shows that CGA suppresses cardiac apoptosis in rats with cardiomyopathy induced by carbon tetrachloride, together with the importance of apoptosis in the formation of DOX-induced cardiotoxicity. In the current study, the caspase-3 elevation was attenuated in the CGA-treated DOX-induced rats, suggesting that the mitochondrial apoptosis pathway could be involved in the inhibitory effect of CGA treatment on DOX-evoked cardiotoxicity.

It has been reported that DT signaling results in the elevation of myocardial protein oxidation and the accumulation of LPO products and results in myocardial oxidative injury [56]. Interestingly, Benzer et al. showed that DOX upregulated DT expression and augmented oxidative stress-related apoptotic mediators, including caspase-3, in cardiac tissue [26]. The current study showed that DT expression is triggered by DOX treatment, which is associated with elevated oxidative stress and high caspase-3 mRNA expression levels. However, CGA markedly inhibited DT expression levels and consequently alleviated DOX-induced myocardial oxidative stress and decreased apoptosis markers.

Although the present report demonstrated CGA’s protective function against DOX-induced myocardial cell death in vivo, CGA still has some limitations. The current study did not perform functional myocardial evaluations, such as an echocardiographic examination or electrocardiogram recording. In many studies evaluating cardiotoxicity in laboratory animals, only a minority of them evaluated the myocardium functionally with the use of echocardiography. In a previous systematic review, it was shown that with respect to ejection fraction quantification, there is rather an accordance to what is considered cardiotoxicity in the evaluation of the myocardial function of laboratory animals [57]. Second, the current study only used male rats to investigate the CGA effects. Therefore, further study of animal (male and female) models of DOX-related cardiotoxicity and, potentially, clinical experiments should be conducted to further investigate CGA’s protective role. Additional limitations to the study are that Bax, Bcl-2, Nrf2, HO-1, and cleaved caspase-3 levels were not measured. mRNA levels do not always coincide with the same protein change, especially for those proteins, such as caspases, that are only active upon cleavage.

## 5. Conclusions

The current study showed, for the first time, that CGA could counteract DOX-induced cardiotoxicity in vivo by inhibiting oxidative stress and decreasing apoptosis markers via mechanisms that involve the activation of the Nrf2/HO-1 signaling pathway and suppressing DT expression. Current study findings suggest that CGA could be a new potential candidate for use in the attenuation of DOX-related cardiotoxicity. Further studies are needed to evaluate the mechanisms involved in the favorable effects of this natural product against DOX-related cardiotoxicity and its clinical applications.

## Figures and Tables

**Table 1:** Histopathological scores of cardiac tissues.

MN cell infiltrations	Absent (0)	
	Mild (1)	<10 MN cells
	Moderate (2)	10–30 MN cells
	Severe (3)	>30 MN cells
Hemorrhage	Absent (0)	
	Mild (1)	<3 hemorrhage focus
	Moderate (2)	3–6 hemorrhage focus
	Severe (3)	>7 hemorrhage focus

**Table 2:** A sequence list of the primers used for RT-PCR.

Genes	Forward Sequence (5′-3′)	Reverse Sequence (3′-5′)
Nfr2	TGTAGTGCGAGGAAGAGGTATGA	GGAGGGAAAGGAGAGGAAGG
HO-1	AAGAGGCTAAGACCGCCTTC	GCATAAATTCCCACTGCCAC
Caspase-3	TGGACAACAACGAAACCTCC	CTCTTGCCTCAGTCATCAGC
GAPDH	GGCACAGTCAAGGCTGAGAATG	ATGGTGGTGAAGACGCCAGTA

**Table 3:** Histopathological findings. * *p* < 0.05 vs. the control group, ^#^ *p* < 0.05 vs. the DOX group.

	Mn Cell Infiltration	Hemorrhage
Control	0.16 ± 0.40	0.16 ± 0.40
CGA	0.33 ± 0.40	0.16 ± 0.40
DOX	2 ± 0.00 *	2.66 ± 0.51 *
DOX + CGA	1.12 ± 0.40 ^#^	1.33 ± 0.51 ^#^

**Table 4:** Scoring of immunohistochemical findings. * *p* < 0.05 vs. the control group, ^#^ *p* < 0.05 vs. the DOX group.

	4-HNE	8-OHdG	DT
Control	0.00 ± 0.00	0.00 ± 0.00	0.00 ± 0.00
CGA	0.00 ± 0.00	0.16 ± 0.40	0.00 ± 0.00
DOX	1.00 ± 0.00 *	2.83 ± 0.40 *	0.83 ± 0.40 *
DOX + CGA	0.16 ± 0.40 ^#^	1.12 ± 0.40 ^#^	0.16 ± 0.40 ^#^

## Data Availability

The datasets used and/or analyzed during the current study are available from the Bilecik Seyh Edebali University, Faculty of Medicine, Department of Pharmacology, upon reasonable and justifiable request, following the rules and procedures of the mentioned university.

## References

[1] Amaral D.G., Witter M.P. (1989). The three-dimensional organization of the hippocampal formation: A review of anatomical data. Neuroscience.

[2] Li X., Liang J., Qu L., Liu S., Qin A., Liu H., Wang T., Li W., Zou W. (2022). Exploring the role of ferroptosis in the doxorubicin-induced chronic cardiotoxicity using a murine model. Chem.-Biol. Interact..

[3] Zhao L., Zhang B. (2017). Doxorubicin induces cardiotoxicity through upregulation of death receptors mediated apoptosis in cardiomyocytes. Sci. Rep..

[4] Sun L., Wang H., Dan X., Yu S., Zhang L., Li X. (2022). Lapatinib induces mitochondrial dysfunction to enhance oxidative stress and ferroptosis in doxorubicin-induced cardiomyocytes via inhibition of PI3K/AKT signaling pathway. Bioengineered.

[5] Bardas E., Arslan Y.K., Polat S., Erisir M., Uslu G.A., Cetin N., Cicek B. (2020). Vitamin E and selenium reduce prednisolone side effects in rat hearts. Int. J. Vitam. Nutr. Res..

[6] Germanakis I., Kalmanti M., Parthenakis F., Nikitovic D., Stiakaki E., Patrianakos A., Vardas P.E. (2006). Correlation of plasma N-terminal pro-brain natriuretic peptide levels with left ventricle mass in children treated with anthracyclines. Int. J. Cardiol..

[7] Christidi E., Brunham L.R. (2021). Regulated cell death pathways in doxorubicin-induced cardiotoxicity. Cell Death Dis..

[8] Abdel-Daim M.M., Khalifa H.A., Ahmed A.A. (2017). Allicin ameliorates doxorubicin-induced cardiotoxicity in rats via suppression of oxidative stress, inflammation and apoptosis. Cancer Chemother. Pharmacol..

[9] Taghizadeh H., Taghizadehghalehjoughi A., Yildirim S., Ozkaraca M., Genc S., Yeni Y., Mokresh M.Y., Hacimuftuoglu A., Tsatsakis A., Tsaroushas K. (2022). Deteriorated Vascular Homeostasis in Hypertension: Experimental Evidence from Aorta, Brain, and Pancreatic Vasculature. J. Pers. Med..

[10] Qi W., Boliang G., Xiaoxi T., Guoqiang F., Jianbo X., Gang W. (2020). Cardamonin protects against doxorubicin-induced cardiotoxicity in mice by restraining oxidative stress and inflammation associated with Nrf2 signaling. Biomed. Pharmacother..

[11] Zhai J., Tao L., Zhang S., Gao H., Zhang Y., Sun J., Song Y., Qu X. (2020). Calycosin ameliorates doxorubicin-induced cardiotoxicity by suppressing oxidative stress and inflammation via the sirtuin 1–NOD-like receptor protein 3 pathway. Phytother. Res..

[12] Cicek B., Genc S., Yeni Y., Kuzucu M., Cetin A., Yildirim S., Bolat I., Kantarci M., Hacimuftuoglu A., Lazopoulos G. (2022). Artichoke (Cynara Scolymus) Methanolic Leaf Extract Alleviates Diethylnitrosamine-Induced Toxicity in BALB/c Mouse Brain: Involvement of Oxidative Stress and Apoptotically Related Klotho/PPARγ Signaling. J. Pers. Med..

[13] Nikitovic D., Juranek I., Wilks M.F., Tzardi M., Tsatsakis A., Tzanakakis G.N. (2014). Anthracycline-dependent cardiotoxicity and extracellular matrix remodeling. Chest.

[14] Bin Jardan Y.A., Ansari M.A., Raish M., Alkharfy K.M., Ahad A., Al-Jenoobi F.I., Haq N., Khan M.R., Ahmad A. (2020). Sinapic acid ameliorates oxidative stress, inflammation, and apoptosis in acute doxorubicin-induced cardiotoxicity via the NF-κB-mediated pathway. BioMed Res. Int..

[15] Bostan H.B., Rezaee R., Valokala M.G., Tsarouhas K. (2016). Cardiotoxicity of nano-particles. Life Sci..

[16] Georgiadis N., Tsaroushas K., Tsitsimpikou C., Vardavas A., Rezaee R., Germanakis I., Tsatsakis A., Stagos D., Kouretas D. (2018). Pesticides and cardiotoxicity. Where do we stand?. Toxicol. Appl. Pharmacol..

[17] Ahiskalioglu A., Ince I., Aksoy M., Ahiskalioglu E.O., Comez M., Dostbil A., Celik M., Alp H.H., Coskun R., Taghizadehghalehjoughi A. (2015). Comparative investigation of protective effects of metyrosine and metoprolol against ketamine cardiotoxicity in rats. Cardiovasc. Toxicol..

[18] Asilaki F., Tsitsimpikou C., Tsarouhas K., Germanakis I., Tzardi M., Kavvalakis M., Ozcagli E., Kouretas D., Tsatsakis A. (2016). Cardiotoxicity in rabbits after long-term nandrolone decanoate administration. Toxicol. Lett..

[19] Refaie M.M., Shehata S., Ibrahim R.A., Bayoumi A.M.A., Abdel-Gaber S.A. (2021). Dose-dependent cardioprotective effect of hemin in doxorubicin-induced cardiotoxicity via Nrf-2/HO-1 and TLR-5/NF-κB/TNF-α signaling pathways. Cardiovasc. Toxicol..

[20] Loboda A., Damulewicz M., Pyza E., Jozkowicz A., Dulak D. (2016). Role of Nrf2/HO-1 system in development, oxidative stress response and diseases: An evolutionarily conserved mechanism. Cell. Mol. Life Sci..

[21] Avci S., Gunaydin S., Ari N.S., Sulukoglu E.K., Erol-Polat O., Gecili I., Yeni Y., Yilmaz A., Genc S., Hacimuftuoglu A. (2022). Cerebrolysin Alleviating Effect on Glutamate-Mediated Neuroinflammation Via Glutamate Transporters and Oxidative Stress. J. Mol. Neurosci..

[22] Nordgren K.K., Wallace K.B. (2020). Disruption of the keap1/nrf2-antioxidant response system After chronic doxorubicin exposure In vivo. Cardiovasc. Toxicol..

[23] Cheng X., Liu D., Xing R., Song H., Tian X., Yan C., Han Y. (2020). Orosomucoid 1 attenuates doxorubicin-induced oxidative stress and apoptosis in cardiomyocytes via Nrf2 signaling. BioMed Res. Int..

[24] Mayer F., Pröpper S., Ritz-Timme S. (2014). Dityrosine, a protein product of oxidative stress, as a possible marker of acute myocardial infarctions. Int. J. Leg. Med..

[25] DiMarco T., Giulivi C. (2007). Current analytical methods for the detection of dityrosine, a biomarker of oxidative stress, in biological samples. Mass Spectrom. Rev..

[26] Benzer F., Kandemir F.M., Ozkaraca M., Kucukler S., Caglayan C. (2018). Curcumin ameliorates doxorubicin-induced cardiotoxicity by abrogation of inflammation, apoptosis, oxidative DNA damage, and protein oxidation in rats. J. Biochem. Mol. Toxicol..

[27] Kwon S.H., Lee H.K., Kim J.A., Hong S.I., Kim H.C., Jo T.H., Park Y.I., Lee C.K., Kim Y.B., Lee S.Y. (2010). Neuroprotective effects of chlorogenic acid on scopolamine-induced amnesia via anti-acetylcholinesterase and anti-oxidative activities in mice. Eur. J. Pharmacol..

[28] Koss-Mikołajczyk I., Todorovic V., Sobajic S., Mahajna J., Geric M., Tur J.A., Bartoszek A. (2021). Natural products counteracting cardiotoxicity during cancer chemotherapy: The special case of doxorubicin, a comprehensive review. Int. J. Mol. Sci..

[29] Abushouk A.I., Ismail A., Abdo-Salem A.M., Afifi A.M., Abdel-Daim M.M. (2017). Cardioprotective mechanisms of phytochemicals against doxorubicin-induced cardiotoxicity. Biomed. Pharmacother..

[30] Naveed M., Hejazi V., Abbas M., Kamboh A.A., Khan G.J., Shumzaid M., Ahmad F., Babazadeh D., FangFang X., Modarresi-Ghazani F. (2018). Chlorogenic acid (CGA): A pharmacological review and call for further research. Biomed. Pharmacother..

[31] Willis M.S., Parry T.L., Brown D.I., Mota R.I., Huang W., Beak J.Y., Sola M., Zhou C., Hicks S.T., Caughey M.C. (2019). Doxorubicin exposure causes subacute cardiac atrophy dependent on the striated muscle–specific ubiquitin ligase MuRF1. Circ. Heart Fail..

[32] Luminari S., Montanini A., Caballero D., Bologna S., Notter M., Dyer M.J.S., Chiappella A., Briones J., Petrini M., Barbato A. (2010). Nonpegylated liposomal doxorubicin (Myocet™) combination (R-COMP) chemotherapy in elderly patients with diffuse large B-cell lymphoma (DLBCL): Results from the phase II EUR018 trial. Ann. Oncol..

[33] Cardinale D., Sandri M.T., Martinoni A., Tricca A., Civelli M., Lamantia G., Cinieri S., Martinelli G., Fiorentini C. (2000). Left ventricular dysfunction predicted by early troponin I release after high-dose chemotherapy. J. Am. Coll. Cardiol..

[34] Chen D., Yu W., Zhong C., Hong Q., Huang G., Que D., Wang Y., Yang Y., Rui B., Zhuang Z. (2022). Elabela ameliorates doxorubicin-induced cardiotoxicity by promoting autophagic flux through TFEB pathway. Pharmacol. Res..

[35] Kwatra M., Kumar V., Jangra A., Mishra M., Ahmed S., Ghosh P., Vohora D., Khanam R. (2016). Ameliorative effect of naringin against doxorubicin-induced acute cardiac toxicity in rats. Pharm. Biol..

[36] Wang D., Tian L., Lv H., Pang Z., Li D., Yao Z., Wang S. (2020). Chlorogenic acid prevents acute myocardial infarction in rats by reducing inflammatory damage and oxidative stress. Biomed. Pharmacother..

[37] Yeni Y., Cakir Z., Hacimuftuoglu A., Taghizadehghalehjoughi A., Okkay U., Genc S., Yildirim S., Saglam Y.S., Calina D., Tsatsakis A. (2022). A selective histamine H4 receptor antagonist, JNJ7777120, role on glutamate transporter activity in chronic depression. J. Pers. Med..

[38] Celebi D., Taghizadehghalehjoughi A., Baser S., Genc S., Yilmaz A., Yeni Y., Yesilyurt F., Yildirim S., Bolat I., Kordali S. (2022). Effects of boric acid and potassium metaborate on cytokine levels and redox stress parameters in a wound model infected with methicillin-resistant Staphylococcus aureus. Mol. Med. Rep..

[39] Genc S., Pennisi M., Yeni Y., Yildirim S., Gattuso G., Altinoz M.A., Taghizadehghalehjoughi A., Bolat I., Tsatsakis A., Hacımüftüoğlu A. (2022). Potential neurotoxic effects of glioblastoma-derived exosomes in primary cultures of cerebellar neurons via oxidant stress and glutathione depletion. Antioxidants.

[40] Bansal N., Adams M.J., Ganatra S., Colan S.D., Aggarwal S., Steiner R., Amdani S., Lipshultz E.R., Lipshultz S.E. (2019). Strategies to prevent anthracycline-induced cardiotoxicity in cancer survivors. Cardio-Oncology.

[41] Sobczuk P., Czerwińska M., Kleibert M., Cudnoch-Jędrzejewska A. (2022). Anthracycline-induced cardiotoxicity and renin-angiotensin-aldosterone system—From molecular mechanisms to therapeutic applications. Heart Fail. Rev..

[42] Bhayana V., Henderson A.R. (1995). Biochemical markers of myocardial damage. Clin. Biochem..

[43] Park K.C., Gaze D.C., Collinson P.O., Marber S.M. (2017). Cardiac troponins: From myocardial infarction to chronic disease. Cardiovasc. Res..

[44] Cheng Y., Wu X., Nie X., Wu Y., Zhang C., Lee S.-Y., Lv K., Leung G.-H., Fu C., Zhang J. (2022). Natural compound glycyrrhetinic acid protects against doxorubicin-induced cardiotoxicity by activating the Nrf2/HO-1 signaling pathway. Phytomedicine.

[45] Zhao L., Qi Y., Xu L., Tao X., Han X., Yin L., Jinyong Peng J. (2018). MicroRNA-140-5p aggravates doxorubicin-induced cardiotoxicity by promoting myocardial oxidative stress via targeting Nrf2 and Sirt2. Redox Biol..

[46] Meng Y.-Y., Yuan Y.-P., Zhang X., Kong C.-Y., Song P., Ma G.-Z., Tang Q.-Z. (2019). Protection against doxorubicin-induced cytotoxicity by geniposide involves AMPKα signaling pathway. Oxidative Med. Cell. Longev..

[47] Zhang X., Hu C., Kong C., Song P., Wu H., Xu S., Yuan Y., Deng W., Ma Z., Tang Q. (2020). FNDC5 alleviates oxidative stress and cardiomyocyte apoptosis in doxorubicin-induced cardiotoxicity via activating AKT. Cell Death Differ..

[48] Li Y., Ren X., Lio C., Sun W., Lai K., Liu Y., Zhang Z., Liang J., Zhou H., Liu L. (2018). A chlorogenic acid-phospholipid complex ameliorates post-myocardial infarction inflammatory response mediated by mitochondrial reactive oxygen species in SAMP8 mice. Pharmacol. Res..

[49] Akila P., Vennila L. (2016). Chlorogenic acid a dietary polyphenol attenuates isoproterenol induced myocardial oxidative stress in rat myocardium: An in vivo study. Biomed. Pharmacother..

[50] Cappetta D., Angelis D.A., Sapio L., Prezioso L., Illiano M., Quaini F., Rossi F., Berrino L., Naviglio S., Urbanek K. (2017). Oxidative stress and cellular response to doxorubicin: A common factor in the complex milieu of anthracycline cardiotoxicity. Oxidative Med. Cell. Longev..

[51] Qin Y., Xie J., Zheng R., Li Y., Wang H. (2022). Oleoylethanolamide as a New Therapeutic Strategy to Alleviate Doxorubicin-Induced Cardiotoxicity. Front. Pharmacol..

[52] Hada Y., Uchida A.H., Otaka N., Onishi Y., Okamoto S., Nishiwaki M., Takemoto R., Takeuchi H., Wada J. (2020). The protective effect of chlorogenic acid on vascular senescence via the Nrf2/HO-1 pathway. Int. J. Mol. Sci..

[53] Li S., Wang W., Niu T., Wang H., Li B., Shao L., Lai W., Li W., Janicki S.J., Wang L.X. (2014). Nrf2 deficiency exaggerates doxorubicin-induced cardiotoxicity and cardiac dysfunction. Oxidative Med. Cell. Longev..

[54] Zheng Y., Li L., Chen B., Fang Y., Lin W., Zhang T., Feng X., Tao X., Wu Y., Fu X. (2022). Chlorogenic acid exerts neuroprotective effect against hypoxia-ischemia brain injury in neonatal rats by activating Sirt1 to regulate the Nrf2-NF-κB signaling pathway. Cell Commun. Signal..

[55] Al-Rasheed N.M., Nawal M., Al-Rasheed L.M., Faddah A.M., Mohamed R.A., Mohammad M.A.-A. (2014). Potential impact of silymarin in combination with chlorogenic acid and/or melatonin in combating cardiomyopathy induced by carbon tetrachloride. Saudi J. Biol. Sci..

[56] Zhang H., Bowen L., Yuhui Y., Yinyi D., Yueting G., Yuncong X., Yanli X., Yonghui S., Guowei L. (2018). Dityrosine administration induces myocardium injury and inflammatory response in mice. Wei Sheng Yan Jiu J. Hyg. Res..

[57] Nikolaos G., Konstantinos T., Ramin R., Haritini N., George E.N., Kass Jean-Lou C.M.D., Dimitrios S., Konstantinos T., Demetrios A.S., Dimitrios K. (2020). What is considered cardiotoxicity of anthracyclines in animal studies. Oncol. Rep..

